# Propeamussiidae, Inoceramidae, and other Bivalvia from the Lower Cretaceous Puez Formation (Valanginian–Cenomanian; Dolomites, South Tyrol, Italy)^☆^

**DOI:** 10.1016/j.cretres.2013.09.002

**Published:** 2013-11

**Authors:** Simon Schneider, James S. Crampton, Alexander Lukeneder

**Affiliations:** a CASP, West Building, 181A Huntingdon Road, Cambridge CB3 0DJ, UK; b GeoZentrum Nordbayern, Paläoumwelt, Friedrich-Alexander-Universität Erlangen-Nürnberg, Loewenichstrasse 28, 91054 Erlangen, Germany; c GNS Science, PO Box 30368, Lower Hutt 5040, New Zealand; d Victoria University of Wellington, PO Box 600, Wellington, New Zealand; e Naturhistorisches Museum Wien, Geologisch-Paläontologische Abteilung, Burgring 7, 1010 Vienna, Austria

**Keywords:** Propeamussiidae, Inoceramidae, Barremian, Tethys, Deep water, Palaeoecology

## Abstract

A bivalve assemblage from the Lower Cretaceous Puez Formation at the type locality, Piz de Puez (Dolomites, South Tyrol, northern Italy) is described. Given the large amount of sedimentary rock screened during the course of this study, the <50 bivalves examined here, although occurring in very low abundance, are considered to represent a reasonably comprehensive sample. The assemblage provides insight into an autochthonous, Mesozoic, deep-water bivalve community, which was dominated by glass scallops. Two species are described as new, *Parvamussium pizpuezense* n. sp. and the giant *P. mordsdrum* n. sp. Presumably, they lived as epifaunal-reclining carnivores and preyed on various meiofauna, occupying a similar ecologic niche as their modern counterparts. Scarce epifaunal, suspension-feeding Inoceramidae entered only by occasional recruitment of larvae into an environment that is inferred to have been characterised by low levels of suspended nutrients.

## Highlights

• An autochthonous Lower Cretaceous deep-water bivalve community is described.
• *Parvamussium* glass scallops are dominant, with a midsize and a giant new species.
• *Parvamussium* achieved deep-water predatory life style during the Early Cretaceous.
• Filter-feeding Inoceramidae settled in the nutrient-poor environment by occasion.

## 1 Introduction

Today, deep-water benthic habitats, i.e. those areas below the euphotic zone at a water depth of more than *c.* 200 m, account for more than 65% of the earth's surface. However, these regions are much more difficult to access than shallow waters and thus, despite considerable effort during the past decades, they remain much less explored. This also appears valid for fossil deep-water environments. Usually, major tectonic movements are needed to expose ancient deep-water sediments at the surface. Because of deposition at great water depths and tectonic displacement, the fossil record in these sediments often suffers from significant diagenetic loss and deformation. Furthermore, due to low primary productivity, nutrients in deep-water settings mostly are scarce and macrobenthic organisms do not form dense populations. As a result, fossils of deep-water organisms commonly occur scattered and in low abundance, and collecting representative samples requires detailed investigation of large rock exposures. During the Mesozoic, ammonites were among the main shell-bearing inhabitants of the neritic zone. Because they evolved comparatively rapidly, ammonites are used widely as biostratigraphic markers and many outcrops of Mesozoic deep-water sediments have thus been sampled extensively. This careful collecting has often yielded representative samples of the rare autochthonous benthic deep-water organisms, which is the case for the bivalves detailed herein.

The Lower Cretaceous deposits of the Puez area in the Dolomites (Southern Calcareous Alps, South Tyrol, Italy) were first sampled for fossils during the late nineteenth century, and the geologists Victor Uhlig (1888) and Haug, 1888, Haug, 1889 published initial descriptions of the geology and fauna of these sediments. During the last decade (2003–2012), the Puez Cretaceous strata were re-sampled intensively by Italian and Austrian scientists, mostly bed by bed. In addition to an impressive number of more than 1200 ammonites, these workers also collected rare echinoids, solitary corals, brachiopods, and bivalves (Lukeneder and Aspmair, 2006, Lukeneder, 2008, Lukeneder, 2010). These bivalves, comprising allochthonous epibionts as well as autochthonous deep-water inhabitants, are described in detail and illustrated herein, and their palaeoecological requirements are discussed.

## 2 Geological setting

**Fig. 1:**
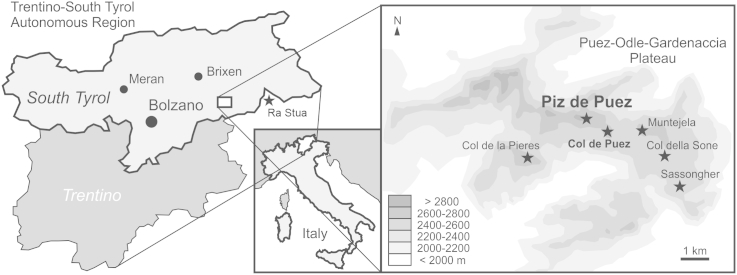
Geographical overview showing the study area and the type locality of the Puez Formation, Piz de Puez. Outcrops of Lower Cretaceous rocks on the Puez-Odle-Gardenaccia Plateau and at Ra Stua locality are indicated by asterisks.

Today, the Lower Cretaceous sediments of the higher Dolomites are preserved as relics, either in pure limestone facies (Biancone Formation; e.g., Mayer and Appel, 1999) or in marl to marly limestone facies (Puez Formation; Lukeneder, 2010). The Puez Formation, preserved predominantly on the Puez-Odle-Gardenaccia Plateau (Fig. 1), was deposited on Triassic Hauptdolomit or Lower Cretaceous Rosso Ammonitico Puezzese limestones (Lukeneder and Aspmair, 2006, Lukeneder, 2010, Lukeneder, 2011). In the Puez area, cone-shaped hills up to 150 m high are formed by the Puez Formation and are overlain by tectonically emplaced slivers of Triassic Dachstein Limestone up to 10 m thick, that have acted as a protection against erosion (Lukeneder and Aspmair, 2006, Lukeneder, 2010).

**Fig. 2:**
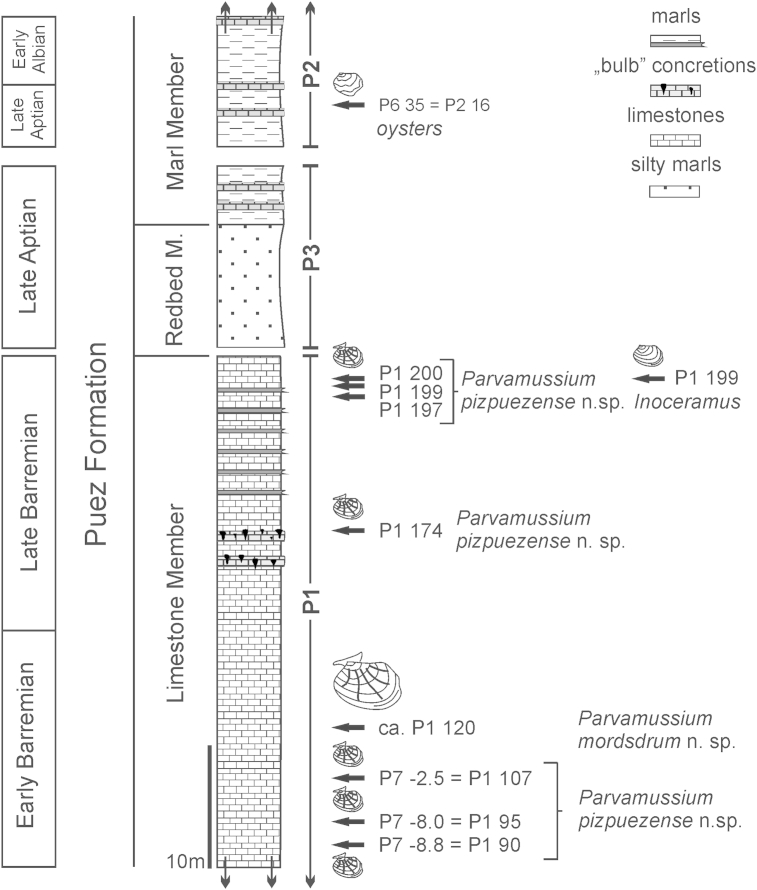
Composite schematic section of the relevant part of the Puez Formation. Levels that have yielded bivalve fossils are indicated. P1 to P7 refer to different sections on the slopes of Piz de Puez, which can be correlated based on lithology and ammonite biostratigraphy.

As described in detail by Lukeneder (2010), the section of the Puez Formation at Puez comprises marls and (marly) limestones that are approximately 120 m thick. The Puez Formation can be subdivided into three members that were deposited during a time span from the Late Valanginian to the Early Cenomanian (Fig. 2). The first, the Puez Limestone Member, consists of approximately 50 m of marly limestones, which are Late Valanginian to Late Barremian in age. The second, the Puez Redbed Member, is only 9 m thick and comprises foraminiferal wackestones to packstones of Aptian age, while the third, the Puez Marl Member, consists of approximately 57 m of alternating marl and limestone beds, dated as Early Aptian to Early Cenomanian. Biostratigraphic data were obtained from ammonoids (Lukeneder, 2012) and nannofossils (E. Halásová, pers. comm., 2012).

The Dolomites (Permian to Late Cretaceous in age) comprise an integral, inner part of the fold-thrust belt (backthrust belt) of the Southern Alps in northern Italy, and emerged as a result of the deformation of the Tethyan passive continental margin of Adria (Bosellini et al., 2003, Castellarin et al., 2006). During the Early Cretaceous, the Dolomites formed part of the Trento Plateau which was situated on the eastern flank of the Lombardian Basin (Bosellini et al., 2003, Lukeneder, 2008), at a palaeolatitude of 20–30° N (Muttoni et al., 2005). The Puez area is located within the Dolomites in the northernmost part of the Trento Plateau. The Cretaceous sediments of the Puez area formed on a submarine plateau, the Puez-Odle-Gardenaccia Plateau. For a more detailed geological overview of the Puez area, refer to Lukeneder, 2010, Lukeneder, 2011, Lukeneder, 2012.

## 3 Material and methods

Most of the specimens described herein derive from systematic bed-by-bed collection of fossils at the Puez locality in the years between 2003 and 2012. With the exception of oysters, which originate from the Late Aptian part of the Puez Marl Member, all specimens have been collected from the Puez Limestone Member and are dated as Early to Late Barremian (Fig. 2). The samples are stored at the Naturhistorisches Museum Wien (NHMW, Vienna). A few additional individuals of *Parvamussium* are available from old, stratigraphically unconstrained NHMW collections and from the holdings of the Naturmuseum Südtirol (Bolzano, South Tyrol, Italy). A single large *Parvamussium* from correlative deposits of the Barremian Puez Limestone Member at the Ra Stua locality north of Cortina d'Ampezzo, Province of Belluno, Veneto is stored at the Museo Paleontologico ‘Rinaldo Zardini’ (Cortina d'Ampezzo, Veneto, Italy). Altogether, only 30 rock samples with bivalves from Piz de Puez have been studied, although some samples contain multiple Inoceramidae and Dimyidae. The bivalves number 43 individuals in total. Due to poor preservation, oysters could not be counted (see details below).

The fossils were prepared mechanically using pneumatic chisels and needles. Most specimens were coated with ammonium chloride prior to photography. Shell details were recorded using a digital microscope camera.

## 4 Systematic palaeontology

Class Bivalvia Linnaeus, 1758
Subclass Pteriomorphia Beurlen, 1944 (emend. Boss, 1982)
Order Myalinida H. Paul, 1939
Superfamily Inoceramoidea C. Giebel, 1852
Family Inoceramidae C. Giebel, 1852
Genus *Inoceramus* J. Sowerby, 1814  
*Type species*. *Inoceramus cuvierii* J. Sowerby, 1814, by monotypy.  
*Remarks*. Inoceramidae have only been mentioned twice, in open nomenclature, in previous studies on the Puez Formation (Haug, 1888, Lukeneder and Aspmair, 2006). The specimen illustrated by Lukeneder and Aspmair (2006) is described below as ?*Neocomiceramus* ex gr. *Neocomiensis*. Whether Haug (1888) recorded ‘*Inoceramus*’ sp., ?*Neocomiceramus* ex gr. *neocomiensis*, or another inoceramid species, remains unclear.

Inoceramids from the early half of the Early Cretaceous are generally poorly known and illustrated, and are commonly described from relatively limited, deformed and incompletely preserved material. Interpretation of many specimens is hampered further by the impact of taphonomic artifacts, whereby different modes of preservation interact with shell structures to produce taxonomically misleading morphologies (Crampton, 2004). For all these reasons, the taxonomy of Early Cretaceous Inoceramidae remains problematic and the large number of nominal taxa is in need of revision. Some important and relevant publications that review or figure Hauterivian to Aptian inoceramids include Pergament, 1965, Pokhialainen, 1969, Pokhialainen, 1972, Pokhialainen, 1985, Glazunova, 1973, Crame, 1985, Dhondt, 1992 and Lazo (2006).  

**Fig. 3:**
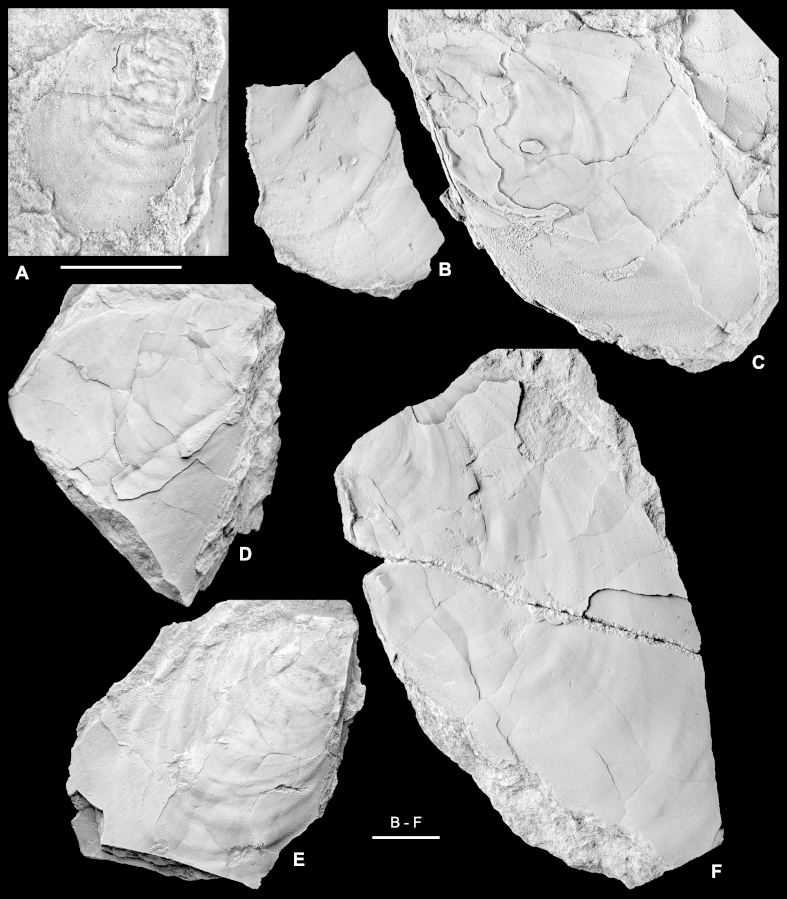
“*Inoceramus*” sp. Piz de Puez; Puez Formation, Puez Limestone Member, level P1 199; late Late Barremian. A. Juvenile right valve with weak regular folds in earliest growth stage, NHMW 2013/0292/0001. B–F. Adult specimens derived from a single rock sample, NHMW 2013/0292/0002–0006. All specimens coated with ammonium chloride. Scale bars: A = 5 mm; B–F = 10 mm.
?1888 *Inoceramus* sp.: Haug, p. 260.  
*Material*. Nine adult specimens derived from a single rock sample (NHMW 2013/0292/0001–0009), and a single juvenile right valve (NHMW 2013/0292/0010). Preserved as flattened internal moulds with patches of the external, prismatic calcite shell layer retained. Upper lower Barremian.  
*Measurements*. Complete specimens: L (max) = 55 mm; H (max) = 52 mm; most specimens are fragmentary; the largest fragment measures approximately 90 mm in height.  
*Description*. Inequivalved; juvenile left valve probably moderately inflated (as judged from flattened material) and becoming weakly inflated with growth; right valve uniformly weakly inflated. Outline obliquely elliptical to ovate, with height exceeding length (descriptive terminology follows Crampton, 1996, fig. 30; Crampton and Gale, 2009, fig. 2). Moderately prosocline; growth axis infracrescent in early growth stages and becoming weakly retrocrescent in adult stages. Adult sculpture of irregularly and widely spaced, very low, rounded, and indistinct commarginal folds. A single juvenile individual (Fig. 3A) has weak but regularly spaced folds restricted to the earliest growth stage (<10 mm). Growth lines very weakly expressed, if at all. Outer prismatic shell thin and nowhere more than 1 mm thick.  
*Remarks*. On present evidence it is not possible to identify the material from the Puez Formation described here to species level. These specimens, although rather nondescript and imperfectly preserved, can be distinguished from most described, elliptical, Hauterivian to early Aptian taxa by their combination of apparently weakly inflated left valves and lack of pronounced commarginal sculpture. Thus, for example, *Coloniceramus colonicus* (Anderson, 1938) and *Heteropteria heteropterus* (Pokhialainen, 1969) (generic assignments of Pokhialainen, 1972) from the Hauterivian to Barremian of Far East Russia and the Pacific coast of North America, have the weak sculpture seen in the Puez Formation material, but possess highly inflated, gryphaeoid umbones in the left valve that project above the hingeline and, in the case of *C. colonicus*, a comparatively thick shell. ‘*Inoceramus*’ *aucella* (Trautschold, 1865) is recorded widely from Hauterivian and Barremian strata of northern mid- to high latitudes and is, again, relatively highly inflated in the left valve and has moderately developed and regularly spaced commarginal folds.  
Genus *Neocomiceramus* Pokhialainen, 1972  
*Type species*. *Inoceramus neocomiensis*d'Orbigny, 1845, by original designation.  
?*Neocomiceramus* ex gr. *neocomiensis* (d'Orbigny, 1845)
Fig. 4.

‘*Inoceramus*’ sp.
Fig. 3A–F.

**Fig. 4:**
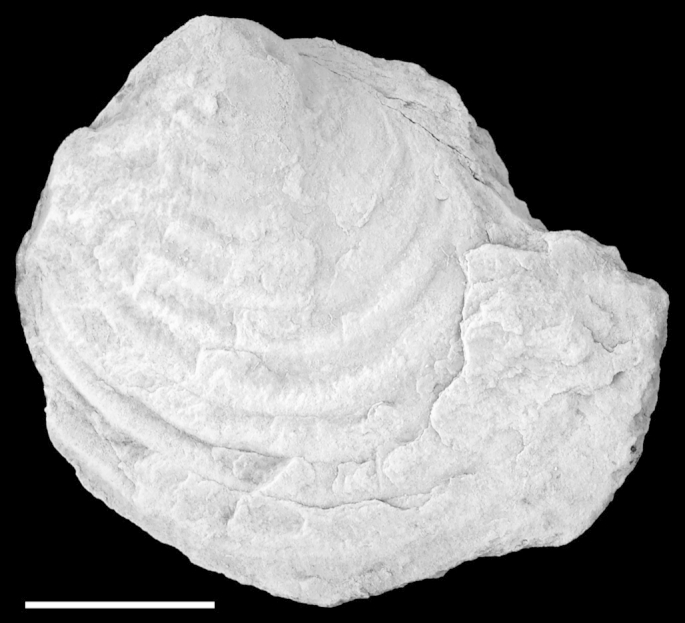
?*Neocomiceramus* ex gr. *neocomiensis* (d'Orbigny, 1845). Piz de Puez; Puez Formation; member and level unknown. Internal mould of a left valve, NHMW 2005z0245/0007. Scale bar = 10 mm.
?1888 *Pholadomya barremensis* Math.: Haug, p. 260.
?1889 *Pholadomya barremensis* Math.: Haug, p. 195.
?1928 *Pholadomya barremensis* Math.: Reithofer, p. 304.
2006 *Inoceramus* sp.: Lukeneder & Aspmair, pl. 8, fig. 10.  
For a more complete synonymy of *N. neocomiensis*, refer to Dhondt and Dieni (1988).  
*Material*. A single internal mould of a left valve (NHMW 2005z0245/0007).  
*Measurements*. L = 32 mm; H = 29 mm.  
*Description*. Left valve moderately weakly inflated. Outline subcircular, weakly prosocline. Sculpture of well-developed, rounded and regularly spaced commarginal folds. Faint radial striations inferred to mark tracks of adductor and/or pallial muscle scars on the internal mould.  
*Remarks*. Haug, 1888, Haug, 1889 and Reithofer (1928), who presumably adopted Haug's identification, reported *Pholadomya barremensis* Matheron from the Puez Formation. Corresponding specimens have not been located in the NHMW collections nor collected during extensive excavations during the last decade. *Neocomiceramus* ex gr. *neocomiensis* and *Pholadomya barremensis* are superficially similar in outline and sculpture, with distinct commarginal ribs. However, the latter species has an additional set of less prominent but distinct radial ribs (not merely faint striations), and it remains unclear whether Haug, 1888, Haug, 1889 confused the two species or not. Thus, we list the respective citations as doubtful synonyms.

The specimen illustrated here shows the characteristic shape and sculpture of the *N. neocomiensis* group, although in the absence of preserved prismatic shell, this identification must be regarded as uncertain. As noted by others, there are a number of widely distributed Valanginian to Aptian species that are apparently closely allied to *N. neocomiensis* (Crame, 1985, Dhondt and Dieni, 1988, Lazo, 2006), and the group may extend downwards into the Berriasian of eastern Europe (*I. belbekensis* Ianin, 1972). Relationships between these nominal taxa remain unclear and the group is in need of careful taxonomic revision. Here, we follow the generic assignment and diagnosis of Lazo (2006).  

**Fig. 5:**
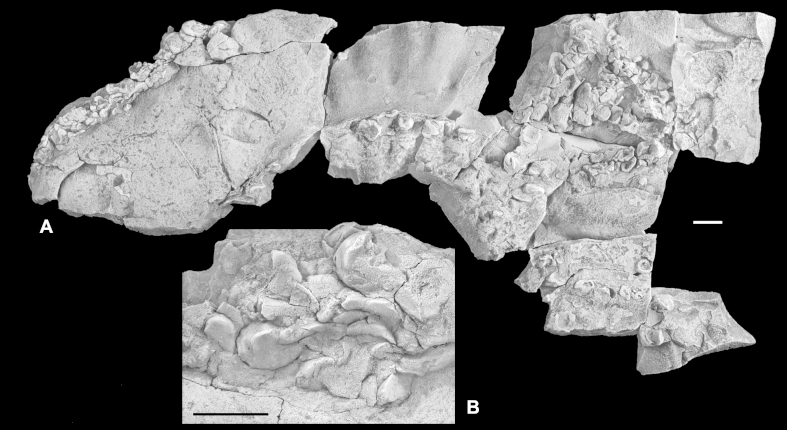
Exogyrinae? indet. Piz de Puez; Puez Formation, Puez Marl Member, level P6 35; Upper Aptian. A. Numerous juvenile specimens attached to the outside of an ammonite mould, NHMW 2013/0292/0011. B. Detail showing smooth, strongly carinate dextrally-coiled shells attached to the venter of the ammonite. Specimen coated with ammonium chloride. Scale bars = 10 mm.
*Material*. A single large, fragmentary mould of an indeterminate ammonite from the Upper Aptian of the Puez Marl Member, with numerous small oyster specimens cemented onto the original outer shell surface of the dissolved cephalopod shell (NHMW 2013/0292/0011).  
*Remarks*. Most of the small specimens (<10 mm) are hardly distinguishable from each other, since they are coated with a thin, encrusting layer of sediment. The aragonitic ammonite shell has dissolved and most of the oyster specimens, formerly attached to the external shell surface, are now incorporated into the external mould of the cephalopod. As a result, the bivalves are mostly visible from the attachment surface only. However, in a few specimens that grew near the venter of the ammonite, the outside of the free (right) valve is exposed. Although a proper identification is impossible, three characters favour an assignment to the Exogyrinae. The free valves are strongly carinate and coiled dextrally, and the shell surface is more-or-less smooth. Moreover, the shells of Exogyrinae typically consist of relatively dark calcite crystals (Nikolaus Malchus, pers. comm., 2012), as can be seen in a few broken specimens from Puez. The oysters cemented onto both lateral sides and the venter of the ammonite, which means that they either colonised the living cephalopod or settled on its empty, floating shell. In either case, they have to be considered allochthonous elements in the fauna.  
Order Pectinida J. Gray, 1854
Suborder Anomiidina J. Gray, 1854
Superfamily Dimyoidea P. Fischer, 1886
Family Dimyidae P. Fischer, 1886
Genus *Atreta* Étallon, 1862  
*Type species*. *Ostrea blandina*d'Orbigny, 1850, by the subsequent designation of Cox (1964).  
*Atreta* sp.
Fig. 6A–D.

Order Ostreida Férussac, 1822
Superfamily Ostreoidea Rafinesque, 1815
Family Gryphaeidae Vialov, 1936
Subfamily Exogyrinae Vialov, 1936  
Exogyrinae? indet.
Fig. 5A–B.

**Fig. 6:**
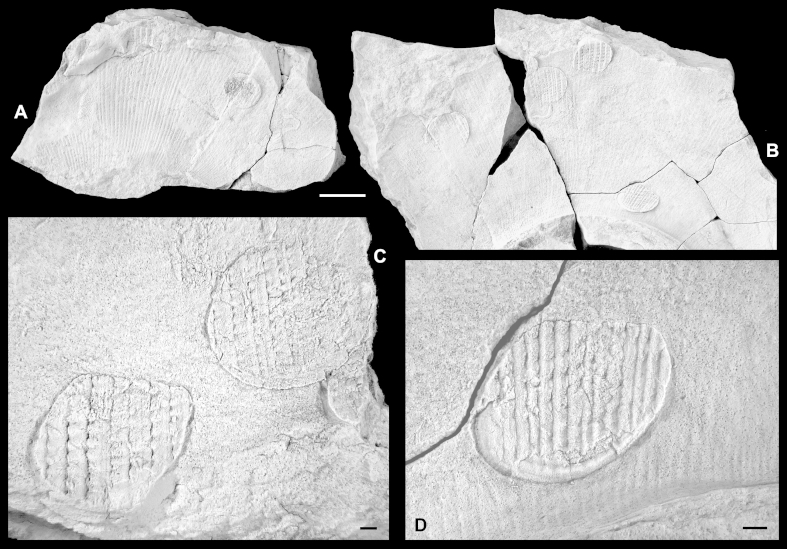
External moulds of ammonites with attached *Atreta* sp., visible from downside view. Piz de Puez; Puez Formation, Puez Limestone Member; Barremian, NHMW 2013/0292/0012–0013. C–D. Details of B. All specimens coated with ammonium chloride. Scale bars: A–B = 10 mm; C–D = 1 mm.

2008 *Placunopsis* sp.: Lukeneder, p. 430.  

*Material*. Fourteen right valves, all preserved on external moulds of ammonites, with only attachment surface visible (NHMW 2013/0292/0012–0025).  
*Measurements*. L (max) = 10 mm; H (max) = 9.5 mm.  
*Description*. Only attachment surface and external rim of right valves visible. Shell obliquely D-shaped in outline, with straight side forming dorsal margin; posterior-ventral shell portion slightly extended. External rim of shell markedly curved upwards (in life position), resulting in shallow, bowl-like shape. Attachment surface basically smooth, with faint commarginal growth lines extending from umbo; umbo positioned slightly below dorsal margin. Several specimens possess prominent pseudo-sculpture of ribs or reticulate pattern, caused by underlying ornament of ammonite shells that acted as substrate (xenomorphism).  
*Remarks*. Unfortunately, only the attachment surfaces of right valves are visible, and the characteristic dimyid attachment scars of the anterior and posterior adductor muscles cannot be observed. Whether the left valves are still attached or not also remains unknown. However, the slightly oblique D-shape of the shells, the umbo, which is positioned significantly below the dorsal shell margin, and the upwardly curved shell rim permit a fairly confident assignment to *Atreta*. In the absence of ornament details of the left valves, a specific attribution is impossible.

All specimens were found cemented to the external surfaces of ammonite shells. As can be seen in the photographs, the specimens themselves show no preferred orientation of their umbones, but they are usually attached to the lower half of the cephalopod shell, when in life position (Lukeneder, 2008). Having presumably been attached to live ammonites, *Atreta* thus was not part of the autochthonous deep-water fauna of the Puez Formation.  

Suborder Entoliidina Hautmann, 2011
Superfamily Entolioidea Teppner, 1922
Family Propeamussiidae Abbott, 1954
Genus *Parvamussium* Sacco, 1897  
*Type species*. *Pecten* (*Pleuronectes*) *duodecimlamellatus* Bronn, 1831, by original designation.  
*Remarks*. *Parvamussium* was proposed as a subgenus of *Amusium* (now assigned to the Pectinidae) by Sacco (1897, p. 48), and distinguished from *Amusium* s. str. by its smaller size and fewer, strong and unpaired internal costae, which do not extend to the shell margin. Sacco (1897) also noted a close relationship between *Parvamussium* and *Propeamussium*, but did not explicitly list similarities or differences. According to Dijkstra and Gofas (2004, p. 37), ‘*Propeamussium* differs from *Parvamussium* by the reduction or absence of the byssal notch on the right valve, by the internal riblets commencing earlier in the ontogeny, and by the gape at the anterior and posterior margins’. All Jurassic to Oligocene propeamussiids that have been studied thoroughly to date belong to *Parvamussium* (Dhondt, 1971, Tamura, 1973, Sundberg, 1989, Waller, 2006); *Propeamussium* did not evolve earlier than the Miocene.

The terminology used below is adopted from Waller, 1986, Waller, 2006. The term ‘antimarginal’ refers to shell elements arranged perpendicular to successive shell margins (Waller, 1986), whereas ‘radial’ denotes straight elements originating from the umbonal region, and meeting shell margins at variable angles during growth. Radial as well as antimarginal elements on the inside and outside of the shell are termed ‘costae’ or, when distinctly narrow, ‘costellae’ herein. Narrow commarginal elements on the inside and outside of the shell are termed ‘lirae’.  

**Fig. 7:**
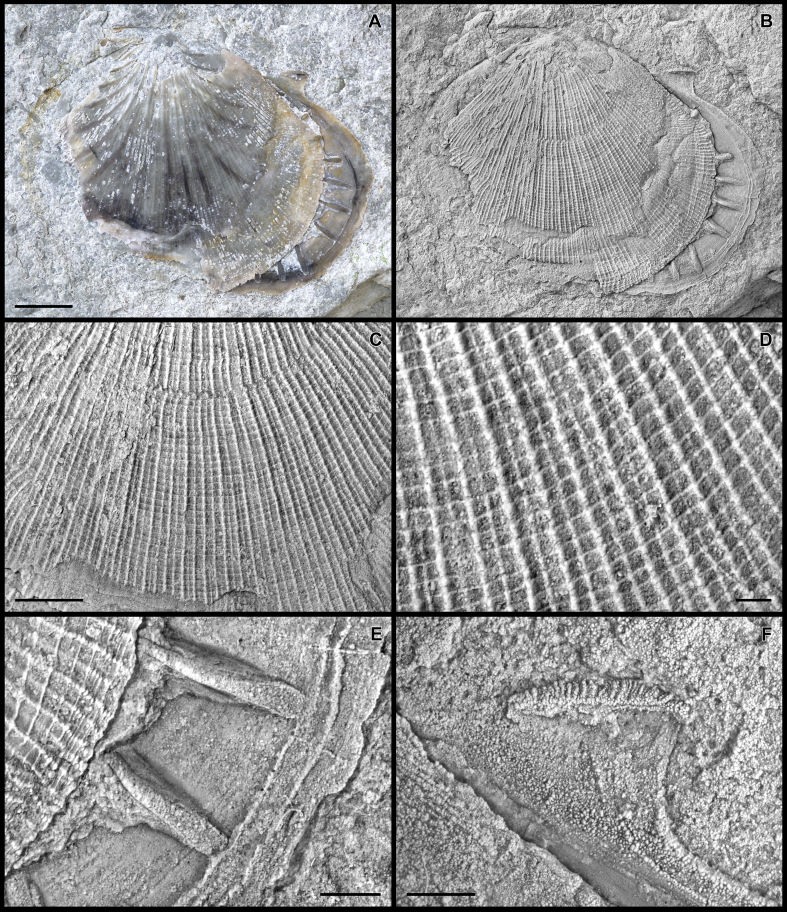
*Parvamussium pizpuezense* n. sp., Holotype. Piz de Puez; Puez Formation, Puez Limestone Member; Barremian, NHMW 2013/0292/0026. A. Double-valved specimen with slightly detached valves in colour, showing semi-translucent shell, with internal costae visible as dark lines. B. Same specimen as in B, coated with ammonium chloride to visualise details of ornamentation. C–D. Details of shell disc in B, showing ornamentation pattern. E. Detail of lower (right) valve in B, showing marginal part of internal costae and commarginal lirae on marginal apron. F. Detail of lower (right) valve in B, showing inside of posterior auricle with resilial teeth. Scale bars: A–C = 5 mm; D = 1 mm; E–F = 2 mm.

**Fig. 8:**
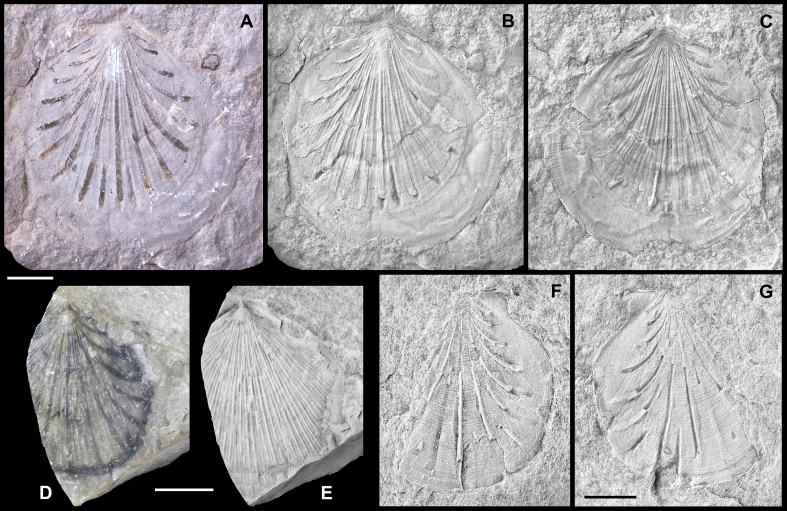
*Parvamussium pizpuezense* n. sp. Piz de Puez; Puez Formation, Puez Limestone Member; Barremian. A–C. Paratype 1. Left valve. A–B. Positive, NHMW 2005z/0245/0004. C. Negative, NHMW 2005z/0245/0027. A. Internal mould with calcitic internal costae transposed onto it. B. Same specimen as in A, coated with ammonium chloride. C. External mould of same specimen as in A–B, coated with ammonium chloride. External ornament is visible from inside. D. Paratype 2. Left valve, showing semi-translucent shell, with internal costae visible as dark lines, NHMW 2013/0292/0027. E. Same specimen as in D, coated with ammonium chloride, showing delicate external ornament. F. Paratype 3. Internal mould of right valve with calcitic internal costae transposed onto it, coated with ammonium chloride. Commarginal external ornamentation is visible as imprint, NHMW 2013/0292/0028a–b. G. Same specimen as in F; inside of shell, coated with ammonium chloride. External ornament is visible from inside. Scale bars = 5 mm.
1888 *Pecten Agassizi* Pict. et Lor.: Uhlig, p. 101.
1888 *Pecten* (*Amussium*) *Agassizi* Pict. & Lor.: Haug, p. 258.
1928 *Pecten Agassizi* Pict. u. Lor.: Reithofer, p. 304.
2006 *Propeamussium* sp.: Lukeneder and Aspmair, pl. 8, figs 6, 9.  
*Diagnosis*. Medium-sized (L < 30 mm) *Parvamussium* with up to 19 internal costae. Shell inaequilateral, length exceeding height. Left valve ornamented with two alternating sets of radial costellae of different strength, crossed by less prominent commarginal lirae, resulting in regular reticulate pattern.  
*Material*. The holotype is an articulated specimen with slightly detached and displaced valves (Fig. 7; NHMW 2013/0292/0026). Paratype 1, a left valve, with internal mould and shell preserved (Fig. 8A–C; internal mould; NHMW 2005z0245/0004 (A–B); shell; NHMW 2005z0245/0027 (C)). Paratype 2, a small fragmentary semi-translucent left valve (Fig. 8D–E; NHMW 2013/0292/0027). Paratype 3, a right valve, with internal mould and shell preserved (Fig. 8F–G; internal mould and shell; NHMW 2013/0292/0028a–b). In addition, six left valves and two right valves have been examined (NHMW 2013/0292/0029–0036).  
*Measurements*. Holotype: L = 26 mm; H = 23 mm; Paratype 1: L = 26 mm; H = 25 mm; Paratype 2 [fragment]: L = 12 mm; H = 17.5 mm; Paratype 3 [fragment]: L = 16 mm; H = 19 mm.  
*Type locality*. Piz de Puez, Dolomites, South Tyrol, Italy.  
*Type stratum*. Puez Limestone Member, Puez Formation; Barremian; the holotype is from the Upper Barremian of horizon P1 174 (see Fig. 2).  
*Derivation of name*. Referring to the name of the type locality, Piz de Puez.  
*Description*. Medium-sized *Parvamussium* (L < 30 mm). Shell fragile, semi-translucent, slightly longer than high, markedly inaequilateral, with extended allometric growth in posterior-ventral direction (Fig. 7, Fig. 8A–C). Dorsal margin straight. Apical angle *c.* 120°. Auricles relatively small, more or less equal sized, with straight margins, meeting at almost right angles. Set of *c.* 25 well-developed resilial teeth visible on inside of posterior auricle in one specimen (Fig. 7F). Prodissoconch unknown.

*Parvamussium pizpuezense* n. sp.
Fig. 7, Fig. 8.

Shell interior ornamented with up to 19 distinctly antimarginal internal costae; 8 costae present at the earliest visible growth stage; additional costae becoming gradually inserted in interspaces with growth (Fig. 8A–C). Internal costae extending down to *c.* 75–80% of shell height. Internal costae often detached from shell and transposed onto internal mould (Fig. 8A–C, F–G). External radial costellae and commarginal lirae of left valve visible as incisions also on inside of shell in central part of disc (Fig. 8C). Numerous minute, slightly undulating radial incisions along inner margin of left valve (Fig. 8C). Inside of marginal apron additionally strengthened with two or three low commarginal lirae (Fig. 7B, E).

Outside of left valve ornamented with up to more than 120 radial costellae and more than 100 commarginal lirae. Radial costellae at each growth stage consisting of two alternating sets; primary costellae twice as high and wide as secondary costellae. With growth, secondary costellae become more prominent, approaching primary costellae in height and strength. At this stage, tertiary costellae become inserted in interspaces between primary and secondary costellae (Fig. 7B–D). Insertion of secondary and tertiary costellae gradual; not coincident in all parts of shell. In late growth stages, all radial costellae attain similar height and strength (Fig. 7B). Commarginal lirae equal to or less prominent than secondary radial costellae. Radial and commarginal interspaces almost equally wide (approximately twice as wide as costellae or lirae), causing very regular reticulate pattern. Internal costae and pallial margin visible from outside of left valve due to semi-translucent shell; often dark reddish-brown in colour (Figs. 7A and 8A).

External surface of right valve and nature of byssal notch, if any, unknown. Ornament of regular commarginal lirae on exterior of right valve visible clearly also from inside in the form of narrow commarginal incisions (Fig. 8F–G).

**Fig. 9:**
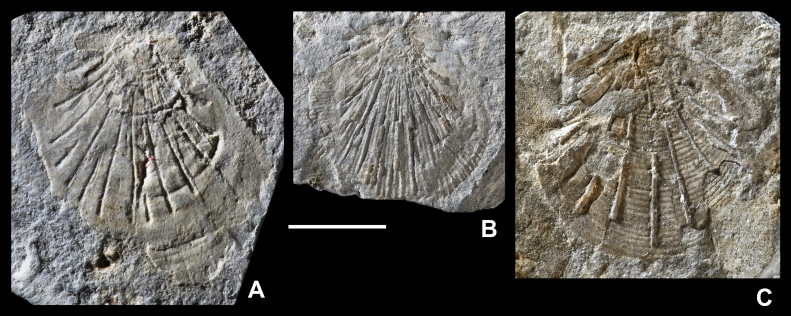
*Parvamussium agassizi* (Pictet and Loriol, 1858). Hauterivian to Barremian, Montagne des Voirons, Haute-Savoie, Rhône-Alpes, southeast France. Muséum d'histoire naturelle de la Ville de Genève; BVIII-44-30933. A. Paralectotype 1, internal mould of left valve, with remnants of shell in central part of disc. Imprints of internal costae are clearly visible (Pictet and Loriol, 1858, pl. 9, fig. 4). B. Lectotype (designated herein), internal mould of left valve with relatively large remnants of shell, showing two alternating sets of sharp radial costellae (Pictet and Loriol, 1858, pl. 9, fig. 2). C. Paralectotype 2, external mould of right valve with remnants of shell, showing internal costae and commarginal ornament (Pictet and Loriol, 1858, pl. 9, fig. 3). Scale bar = 5 mm.

*Remarks*. Compared to most extant and fossil species of *Parvamussium*, *P. pizpuezense* n. sp. is of medium size, as are most Mesozoic representatives of the genus. *Parvamussium agassizi* (Pictet and Loriol, 1858) (Fig. 9A–C; Muséum d'histoire naturelle de la Ville de Genève; BVIII-44-30933) is probably the most closely related congener from the Hauterivian to Barremian of the Montagne des Voirons (Haute-Savoie, Rhône-Alpes, southeast France). Thus, it corresponds well, both with regard to geological age and palaeogeography, with specimens from Piz de Puez. Both species are similar in shape and number of internal antimarginal costae. However, *P. agassizi* differs significantly from *P. pizpuezense* n. sp. in the ornamentation of the left valve. The specimen figured by Pictet and Loriol (1858) on plate 9, figure 2, which is herein designated lectotype (see Fig. 9B), is much smaller than the holotype of *P. pizpuezense* n. sp. It obviously is an internal mould with remnants of shell, providing, however, a relatively good impression of external morphology. *Parvamussium agassizi* is ornamented with approximately 50 radial costellae, which occur in two alternating sets of different strength, just as in *P. pizpuezense* n. sp., and even correspond in number to the appropriate growth stage in the latter. However, in *P. agassizi* the costellae are much broader than in *P. pizpuezense* n. sp., with narrow interspaces and sharp crests. Most significantly, however, the left valve of *P. agassizi* lacks any commarginal ornamentation.

Two species from the Berriasian of the Crimea Peninsula (Ukraine; Arkadiev and Bogdanova, 2012) that presumably belong to *Parvamussium* were first described by Retowski (1893). *Pecten* (*Amusium*) *pawlovi* Retowski, 1893 clearly differs from *Parvamussium pizpuezense* n. sp. in its significantly higher number of internal costae, >22 according to Retowski (1893) and 33–34 according to Arkadiev and Bogdanova (2012). *Pecten* (*Amusium*) *sokolowi* Retowski, 1893 is slightly smaller than *Parvamussium pizpuezense* n. sp. and has almost straight radial internal costae, whereas they are distinctly antimarginal in the new species. Moreover, *Pecten* (*Amusium*) *sokolowi* has only up to 70 external radial costellae and much weaker commarginal lirae than *P. pizpuezense* n. sp.

As far as we are aware, no additional species of *Parvamussium* have been described from Tethyan Cretaceous rocks in Europe. The Turonian to Maastrichtian boreal species *Parvamussium inversum* (Nilsson, 1827) reaches only up to 8 mm in length and height, and is thus distinctly smaller than *P. pizpuezense* n. sp. Moreover, it lacks radial ornamentation on the left valve (Griepenkerl, 1889, Dhondt, 1971).

Johnson (1984) revised the Jurassic Propeamussiidae from Europe and adjacent regions and recognised three species, which he assigned to the genus *Propeamussium*; however, that author did not discuss the relationships between *Propeamussium*, *Parvamussium* and allied genera. From his descriptions, however, it becomes clear that the Jurassic species all retain at least a weak byssal notch in adulthood and thus belong to *Parvamussium* according to the taxonomic concept applied herein. This observation is supported by Waller (2006), although he regarded the Early to Middle Jurassic representatives of the Propeamussiidae as intermediate between the Triassic genus *Filamussium* Waller, 2006 and the Late Jurassic to Recent *Parvamussium*, based on differences in the ordering of calcite prisms. Since Waller (2006) did not establish a separate genus for Early to Middle Jurassic taxa, we herein continue to treat the respective species as representatives of *Parvamussium*.

*Parvamussium pizpuezense* n. sp. differs from the three Jurassic species in being longer than high, in its inaequilateral shell and in its left valve that is more densely ornamented with more than 100 radial costellae and commarginal lirae. Additionally, the Late Pliensbachian to Early Bajocian (see Johnson, 1984) *Parvamussium laeviradiatum* (Waagen, 1868) clearly differs from all other congeners in its dorsally extended, horn-like auricles in the right valve. This species was placed in a separate genus, *Varientolium*, by Andreeva (1966). *Parvamussium laeviradiatum* and the Late Pliensbachian to Bathonian *P. pumilum* (Lamarck, 1819) also differ from *P. pizpuezense* n. sp. in having no more than 13 near-straight radial internal costae; no additional internal costae are intercalated. The Late Jurassic (Late Oxfordian to Early Tithonian) *Parvamussium nonarium* (Quenstedt, 1857) has slightly antimarginal internal costae and intercalary costae, but differs from *P. pizpuezense* n. sp. in being much smaller, and in having much coarser reticulate ornamentation on the left valve. An additional species not mentioned by Johnson (1984), *Pecten spendiarovi* Abel, 1897, from the Kimmeridgian to Tithonian Klentnice Beds of northeast Austria (Schneider et al., 2013), probably also belongs to *Parvamussium*. It is much more slender than *P. pizpuezense* n. sp. and has radial external costellae on both valves, according to the drawings of Abel (1897); the holotype of *P. spendiarovi* could not be traced in Vienna, and may be lost.

Several Mesozoic species of *Parvamussium* from the circum-Pacific area (California, Japan, Indonesia, Thailand) were discussed in detail by Sundberg (1989) and Saengsrichan et al. (2011). These all are distinctly smaller than *P. pizpuezense* n. sp. and differ in the number and arrangement of internal costae and external disc ornamentation. They are thus not explicitly considered herein.  

**Fig. 10:**
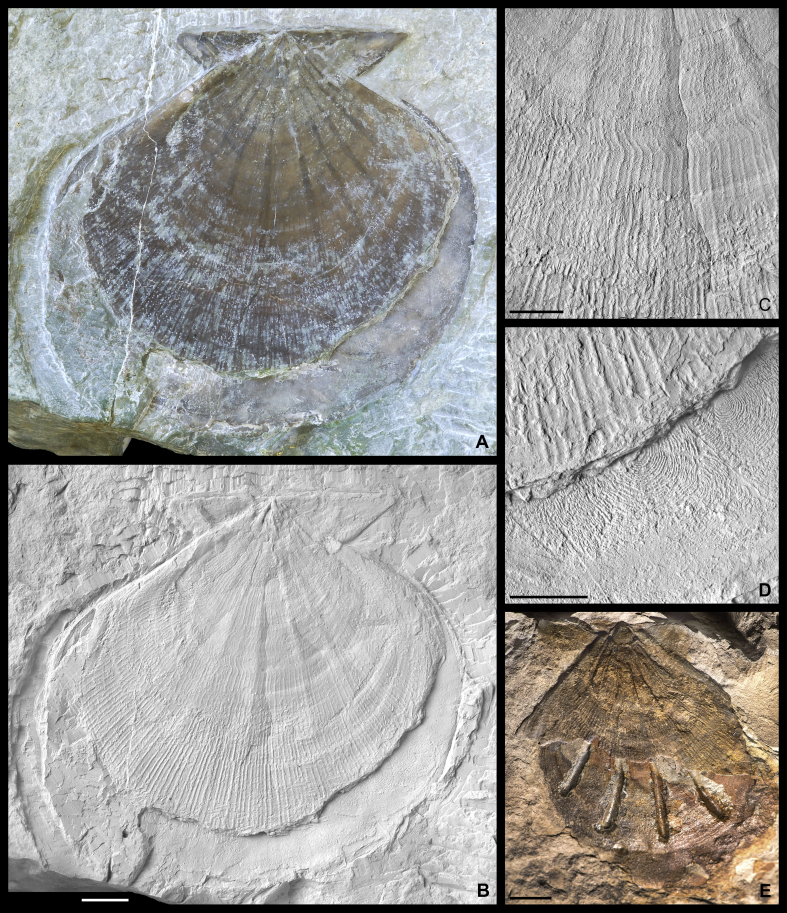
*Parvamussium mordsdrum* n. sp. A–D. Holotype. Piz de Puez; Puez Formation, Puez Limestone Member; late Early Barremian. Articulated specimen, NHMW 2013/0292/0037. A. Colour photograph, showing semi-translucent shell, with internal costae visible as dark lines. B. Same specimen as in A, coated with ammonium chloride; the numerous undulating radial costellae are clearly visible. C. Detail of B, showing delicate ornamentation. D. Detail of B, showing broken margin of left valve and inside of marginal apron of right valve with beekite rings. E. Paratype. Ra Stua; Puez Formation, Puez Limestone Member; late Early Barremian. Museo Paleontologico ‘Rinaldo Zardini’, Cortina d'Ampezzo, Veneto, Italy, no. 1868 RS-Z. Scale bars = 10 mm.
*Diagnosis*. Extraordinarily large *Parvamussium* species (L > 90 mm) with 13 internal costae extending from umbo to approximately 70% of shell height.  
*Material*. Holotype, a large articulated specimen from the upper Lower Barremian of the Puez Limestone Member at Piz de Puez (NHMW 2013/0292/0037). Paratype, a medium-sized left valve from the same unit at Ra Stua, north of Cortina d'Ampezzo, Belluno Province, Veneto (Museo Paleontologico “Rinaldo Zardini”; no. 1868 RS-Z).  
*Measurements*. Holotype: L = 92 mm; H = 87 mm; Paratype: L = *c.* 65 mm; H = *c.* 63 mm [paratype measurements based on photograph].  
*Type locality*. Piz de Puez, Dolomites, South Tyrol, Italy and Ra Stua, north of Cortina d'Ampezzo, Province of Belluno, Veneto, Italy.  
*Type stratum*. Puez Limestone Member, Puez Formation; late Early Barremian.  
*Derivation of name*. Derived from the Bavarian–Austrian vernacular words ‘mords Drum’, meaning ‘very large specimen’.  
*Description*. Shell exceptionally large (L (max) = 92 mm), semi-translucent; slightly longer than high; anterior, ventral and posterior shell margins well rounded, forming a subcircular segment; dorsal shell margin straight. Anterior-dorsal and posterior-dorsal disc margins almost straight. Apical angle *c.* 115°. Auricles comparably large, almost perfectly trigonal; posterior auricle significantly larger than anterior one. Posterior auricle ornamented with growth lines, becoming more prominent, almost plicate, just below the dorsal margin. Prodissoconch not preserved.

*Parvamussium mordsdrum* n. sp.
Fig. 10A–E.

Shell interior ornamented with initially seven (?) and finally up to 11 near-straight radial internal costae in adulthood. New costae inserted across anterior and posterior parts of shell during ontogeny. Outside of left valve ornamented with up to *c.* 120 slightly irregular, sinuous, radial costellae. Earlier growth stage ornamented with *c.* 60 costellae only; additional set of costellae gradually but almost simultaneously emerging from shell surface at shell height of *c.* 55 mm. No commarginal lirae present; growth lines irregular, faint. Marginal apron broken and largely dissolved. Posterior and central portions of dissolved apron removed during preparation. Outside of right valve, including potential byssal notch, unknown.  

**Fig. 11:**
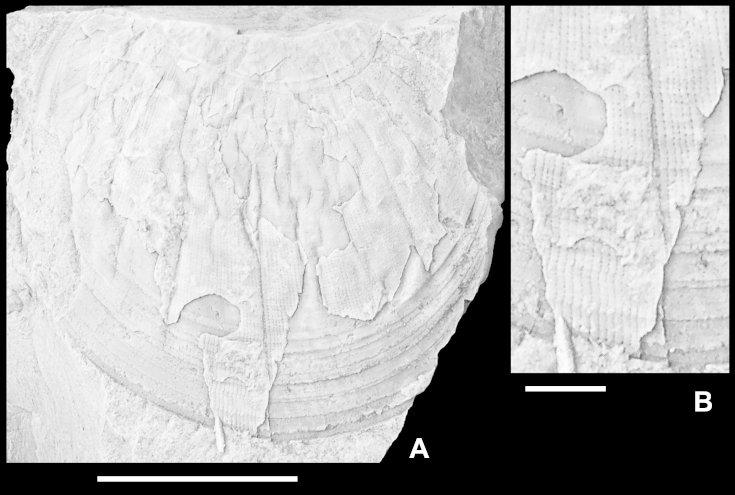
Pectinida indet. Piz de Puez; Puez Formation, Puez Limestone Member; Barremian, NHMW 2013/0292/0038. A. Fragment of disc with remnants of shell, coated with ammonium chloride; showing regularly spaced radial incisions and punctuate radial lines. B. Detail of A. Scale bars: A = 10 mm, B = 2 mm.
*Material*. A single disc fragment with remnants of shell from the Barremian Puez Limestone Member (NHMW 2012/0292/0038).  
*Measurements*. L = 23 mm; H = 21 mm.  
*Description*. Fragmentary specimen with subcircular disc. Shell exterior with nine, equally-spaced, narrow radial incisions obviously not corresponding to internal costae. Shell ornamented with numerous (>150) slightly sinuous radial lines of regularly spaced, minute pits. Irregular growth lines visible mainly on internal mould.  
*Remarks*. Since major portions of the shell, including the entire hinge region, are lacking, a comprehensive description and accurate identification are impossible. Certainly, the specimen belongs to a distinct, third species of pectinid affinities.  
Order, family and genus indet.
Pteriomorphia indet.  
*Material*. Three indeterminate shell fragments of different bivalves (NHMW 2013/0292/0039–0041).  
*Remarks*. Obviously all three shells were originally of calcitic mineralogy, making a placement within the Pteriomorphia very likely. Due to the poor preservation, confident identifications are impossible.  
Subclass, order, family and genus indet.
Bivalvia indet.
Fig. 12.

*Remarks*. The unique combination of the most conspicuous features of *Parvamussium mordsdrum* n. sp., i.e. its extraordinary size and the ornamentation of *c.* 120 costellae, clearly distinguish this species from all other Jurassic and Cretaceous species of the genus.  
Suborder, family and genus indet.
Pectinida indet.
Fig. 11A–B.

**Fig. 12:**
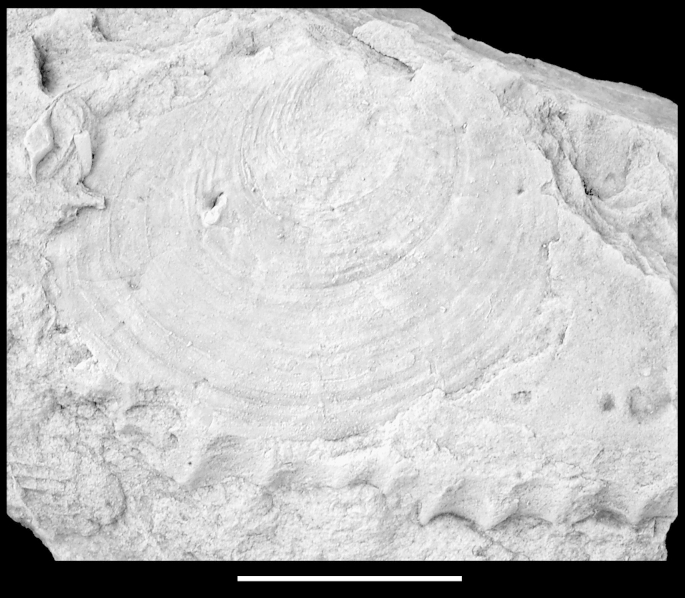
Bivalvia indet. Piz de Puez; Puez Formation, Puez Limestone Member; Barremian, NHMW 2013/0292/0042. External mould preserved inside a fragmentary ammonite; specimen coated with ammonium chloride. Scale bar = 10 mm.
*Material*. A single external mould of a left (?) valve (NHMW 2013/0292/0042).  
*Measurements*. L = 23 mm; H = 19 mm.  
*Description*. Shell subcircular in outline, with slight postero-dorsal slope and faintly indicated postero-ventral flexure; almost flat. Umbonal region poorly preserved. Shell smooth with distinct, regular, isometric growth lines.  
*Remarks*. It seems that this specimen is barely compressed, since the growth lines appear regular and undistorted. Unfortunately, the umbonal region is poorly preserved. Obviously the specimen did not possess calcitic shell portions, since calcitic shells, even those of the fragile, translucent Propeamussiidae, are perfectly preserved in the Puez sediments, whereas aragonite is entirely dissolved. The specimen is preserved inside the body chamber of a specimen of the ammonite *Phyllopachyceras ladinum*.

## 5 Discussion

The type locality of the Puez Formation has been sampled intensely during the last 10 years. Given that more than 1200 ammonite specimens have been collected over this period (Lukeneder and Aspmair, 2006, Lukeneder, 2008), the fact that fewer than 50 bivalves have been recovered seems surprising. Furthermore, only a part of the bivalve fauna from the Puez Formation comprises autochthonous elements. Even these individuals belong to several successive benthic communities of Early to late Late Barremian age. However, as already outlined above, population densities of deep-water bivalves commonly are low and our relatively comprehensive collection from the Puez Formation provides a rare opportunity to study such a fauna.

*Allochthonous elements*. The calcitic shells of *Atreta* and the small oysters were cemented to the external surface of aragonitic ammonite shells that are now dissolved. Thus, we cannot tell whether the bivalves settled on live or dead ammonites. For the oysters, which attached on both sides and on the venter of the ammonite shell, it seems likely that they cemented to a live or at least floating cephalopod. Moreover, *Atreta* and the oysters were filter feeders and needed a considerable amount of suspended organic matter to survive. The near-total absence of filter-feeding organisms from the bottom community, however, suggests that bottom waters were nutrient depleted.

*Autochthonous elements*. Inoceramid palaeobiology remains poorly understood, but these bivalves were apparently distributed widely from inner shelf to abyssal depths, although it seems that they were probably most abundant close to continental landmasses and oceanic islands (Crampton, 1996 and references therein). They were epifaunal to semi-infaunal suspension feeders that could occupy a wide range of substrate types. Obliquely elongate forms, such as *Inoceramus* sp. from the Puez Formation, may have been semi-infaunal, whereas more circular taxa are likely to have been fully epifaunal recliners (Crampton, 1996). Some species are likely to have been byssate, at least at early growth stages, although it is not known whether this was the case for the taxa described here. Many inoceramids were apparently tolerant of dysaerobic conditions (e.g., Sageman, 1989), which explains their presence in some strata lacking other shelly faunas. However, there is no evidence for dysaerobic conditions from those layers of the Puez Formation that have yielded bivalve fossils.

Propeamussiids occupy an entirely exceptional position with regard to feeding type among bivalves. They have adapted to a carnivorous life style, preying on Copepoda (Hicks and Marshall, 1985), Foraminifera, and different kinds of eggs and larvae (Morton and Thurston, 1989). This is reflected in a small, reduced stomach, and strongly modified gills that have lost their filtering capacity (Morton and Thurston, 1989, Waller, 2006, Mikkelsen and Bieler, 2008). Most extant species in the Propeamussiidae inhabit bathyal or abyssal zones (Knudsen, 1970, Mikkelsen and Bieler, 2008), and their carnivorous life style clearly represents an adaptation to such environments in which suspended nutrients are usually scarce. Apart from the Propeamussiidae, only some Poromyida, which thrive predominantly in similar habitats, have developed carnivorous feeding strategies (e.g., Knudsen, 1979, Poutiers and Bernard, 1995). Related to their feeding habits, most propeamussiids, including *Parvamussium*, are free-living recliners, capable of swimming (Waller, 2006).

The shell of the propeamussiid right valve consists of an inner layer of crossed lamellar aragonite, a very thin middle layer of lathic or foliated calcite, and an outer layer of columnar prismatic calcite. In contrast, the shell of the left valve is composed of only two layers, i.e. an inner layer of crossed-lamellar aragonite and an outer layer of foliated calcite (Waller, 2006). The internal costae in both valves consist of lathic calcite and are embedded in the aragonitic inner layer. A major part of the hinge elements is also composed of aragonite. This complex microstructure pattern explains the unique preservation of some of the specimens described above. In most specimens, aragonite obviously was dissolved during diagenesis and hinge structures are no longer preserved. Furthermore, the internal costae became detached from the remainder of the shell, and are now commonly transposed onto the internal mould, simply because of a better adhesion to the marly sediment than to the smooth interior of the calcitic shell (Fig. 8A–C, F–G). Moreover, the external ornament of the shells can be traced from interior view mainly because of the lack of the aragonitic shell portions (Fig. 8C). The shape and arrangement of the internal costae varies from almost straight and radial as seen in *Parvamussium mordsdrum* n. sp., to distinctly antimarginal as in *P. pizpuezense* n. sp. To date, it is unclear whether this feature is of any phylogenetic or functional significance. However, new internal costae are formed at the anterior and posterior ends of the disc in species with straight internal costae, whereas they are usually inserted between existing costae in species with antimarginal costae. Moreover, the growth of radial *vs* antimarginal costae must have involved different modes of shell morphogenesis, as has been suggested for shell folds by Crampton and Gale (2009 and references therein).

The fossil record of the Propeamussiidae starts in the Middle to Late Triassic (Hautmann, 2001, Waller, 2006; a Carboniferous first occurrence date was proposed by Mikkelsen and Bieler 2008, but has not been supported by data) and, despite the relative scarcity of well-preserved deep-water assemblages, is largely continuous through all stages to the present day. The great majority of propeamussiids has been recorded from (fossil) bathyal environments (e.g., Löffler, 1999, Dijkstra and Gofas, 2004, Del Río et al., 2008, Dijkstra and Maestrati, 2008, Dijkstra and Maestrati, 2010, Harzhauser et al., 2011), although the family exhibits a total depth range from the intertidal zone down to *c.* 5000 m (Waller, 2006).

It has been proposed that the Propeamussiidae thrived in shallow neritic environments until the Early Cenozoic and subsequently retreated to greater water depths (Damborenea, 2002, Del Río et al., 2008). Evidence from the Puez Formation of the Dolomites (Piz de Puez and Ra Stua) as well as from the Montagne des Voirons (Pictet and Loriol, 1858), however, suggests that several species of *Parvamussium* already were adapted to life at greater water depths during the Early Cretaceous.

Cenozoic records of propeamussiids from shallow waters should be regarded with caution, since they may have come from regions that are subject to vigorous coastal upwelling, as has been suggested for Lower Pliocene deposits at Velerín-Antena and Velerín-Carretera (Estepona region, southern Spain; Aguirre et al., 2005) that have yielded abundant propeamussiids (Lozano Francisco, 1997, Studencka et al., 2012); thus, these records may represent exceptions from the rule.

Different maximum sizes for members of the Propeamussiidae are reported in the literature. Waller (2006) and Mikkelsen and Bieler (2008) noted general sizes of up to 120 mm, but did not cite any particular species or references. Huber (2010) indicated a maximum shell height of 90 mm, measured for the Recent *Propeamussium dalli* (E.A. Smith, 1885). Regardless of precise upper limits, it is clear that *Parvamussium pizpuezense* n. sp. is medium sized, whereas *Parvamussium mordsdrum* n. sp., reaching 92 mm in length, clearly ranges into the top size class for the family. Obviously, evolutionary size increase in the sense of Cope's Rule is not the driving force of gigantism in propeamussiids, since exceptionally large taxa occur throughout the evolutionary history of the group (Waller, 2006). More likely, specific environmental factors, which remain unknown, may have promoted evolution of large species.

In total, a single individual of *P. mordsdrum* n. sp. and 16 specimens of *Parvamussium pizpuezense* n. sp. from the Puez Formation at Piz de Puez have been collected (including those stored at Naturmuseum Südtirol, Bolzano, which were not examined in detail for the present study). Thus, Propeamussiidae account for a major portion of the autochthonous benthic fauna of the Puez Formation. Coincidentally, no shallow-water taxa are recorded from the respective sediments, which may clearly indicate deposition at significant depths. The large individual of *P. mordsdrum* n. sp. provides indirect evidence of a rich meiofauna (potentially including inoceramid larvae), which generally is the preferred prey of the glass scallops.

In addition to bivalves, the autochthonous fauna of the Puez Formation comprises benthic Foraminifera, Scleractinia (*Cycloseris*?), Serpulidae (*Glomerula*?), Ostracoda, Brachiopoda (*Pygope*, *Triangope*), Ophiuroidea and Echinoidea (Lukeneder and Aspmair, 2006, Lukeneder, 2008). Discoid solitary corals of the genus *Cycloseris*? settled exclusively on dead ammonite shells and achieved a maximum diameter of more than 40 mm; such corals are inferred to have lived for several years (Lukeneder, 2008; compare also Hamel et al., 2010). This implies very low sedimentation rates at least during the lifetime of the corals. Similar conditions can be deduced from pygopid brachiopods, which are usually also inferred to have favoured low-energy, deep-water environments with low sedimentation rate (Kázmér, 1993, Lukeneder, 2002; compare also Vörös, 2005). The peculiar assemblage of irregular echinoids is currently under study.

## 6 Conclusions

Bivalvia from the Lower Cretaceous Puez Formation at the type locality, Piz de Puez (Dolomites, South Tyrol, northern Italy), occur in very low abundance. Given the amount of sedimentary rock screened over the past decade, however, the bivalves examined here, numbering fewer than 50, are inferred to represent a reasonably comprehensive sample of the once-living fauna. Small oysters and *Atreta* have been found attached to the outside of ammonite shells and are interpreted to be allochthonous elements. However, the collection also provides insight into an autochthonous Mesozoic deep-water bivalve community, which is dominated by glass scallops of the genus *Parvamussium*. The abundant *Parvamussium pizpuezense* n. sp. and the rare, giant *P. mordsdrum* n. sp. presumably were adapted to low-nutrient deep-water conditions, living as epifaunal-reclining carnivores and preying on various meiofauna. Scarce epifaunal, suspension-feeding Inoceramidae probably indicate occasional recruitment of larvae into an environment with low levels of suspended organic matter. The two new species of *Parvamussium* from the Dolomites and the contemporary *P. agassizi* (Pictet and Loriol) from southern France clearly show that several members of this genus had adapted to deep-water environments as early as the Early Cretaceous, and thus occupied a similar ecological niche to their modern counterparts.
